# Using equitable impact sensitive tool (EQUIST) and knowledge translation to promote evidence to policy link in maternal and child health: report of first EQUIST training workshop in Nigeria

**DOI:** 10.11604/pamj.2017.28.37.13269

**Published:** 2017-09-14

**Authors:** Chigozie Jesse Uneke, Issiaka Sombie, Henry Chukwuemeka Uro-Chukwu, Ermel Johnson, Friday Okonofua

**Affiliations:** 1 African Institute for Health Policy and Health Systems Studies, Ebonyi State University, PMB 053 Abakaliki, Nigeria; 2 Organisation Ouest Africaine de la Santé, 175, Avenue Ouezzin Coulibaly, 01 BP 153 Bobo-Dioulasso 01, Burkina Faso; 3 Women's Health and Action Research Centre Benin City Nigeria

**Keywords:** EQUIST, knowledge translation, policymakers, researchers, evidence-informed, capacity

## Abstract

The Equitable Impact Sensitive Tool (EQUIST) designed by UNICEF and knowledge translation (KT) are important strategies that can help policymakers to improve equity and evidence-informed policy making in maternal, newborn and child health (MNCH). The purpose of this study was to improve the knowledge and capacity of an MNCH implementation research team (IRT) and policy makers to use EQUIST and KT. A modified “before and after” intervention study design was used in which outcomes were measured on the target participants both before the intervention (workshop) is implemented and after. A 5-point likert scale according to the degree of adequacy was employed. A three -day intensive EQUIST and KT training workshop was organized in Edo State, Nigeria with 45 participants in attendance. Some of the topics covered included: (i) Knowledge translation models, measures & tools; (ii) Policy review, analysis and contextualization; (iii) Policy formulation and legislation process; (iv) EQUIST Overview & Theory of change; (v) EQUIST's situation analysis, scenario analysis and scenario comparison. The pre-workshop mean of understanding of use of KT ranged from 2.02-3.41, while the post-workshop mean ranged from 3.24-4.30. Pre-workshop mean of understanding of use of EQUIST ranged from 1.66-2.41, while the post-workshop mean ranged from 3.56-4.54 on the 5point scale. The percentage increase in mean of KT and EQUIST at the end of the workshop ranged from 8.0%-88.1% and 65.6%-158.4% respectively. Findings of this study suggest that policymakers' and researchers KT and EQUSIT use competence relevant to evidence-informed policymaking can be enhanced through training workshop.

## Introduction

Throughout the world especially in low and middle-income countries (LMICs), policymakers often face difficult decisions about how to allocate scarce resources, balancing equity (eliminating inequality in health outcomes between groups), effectiveness (maximizing results for the country as a whole) and efficiency (making the most rational use of resources) [1]. In many LMICs, including Nigeria, among the most important health policy areas where decision making is increasingly becoming challenging are those related to maternal, newborn and child health (MNCH). In Nigeria, maternal and child health outcome is reportedly poor with more than 1 million newborn, infant, and child deaths and more than 50,000 maternal deaths every year [2, 3]. However, in recent years there has been a noteworthy reduction in maternal and child mortality in Nigeria. The national maternal mortality ratio reduced from 800/100,000 in 2005 [4] to 576/100,000 in 2013 [5], while the under-five mortality rate (U5MR) reduced from 201 per 1000 live births in 2003 [6, 7] to 117 per 1000 live births in 2013 [8]. It is pertinent to state that the decrease in U5MR in Nigeria and in most LMICs for instance has been accompanied by increased inequity in health outcomes between the poor and those better off [9]. This explains why the United Nations Children's Fund (UNICEF) argues for abandoning the “mainstream approach” where scaling-up of child health interventions is first provided to more readily accessible (and typically wealthier) groups in society. Instead, an “equity-focused” approach is suggested, contending that it is more cost-effective to target interventions at the poorest in society, resulting in a greater U5MR decrease while also positively impacting upon equity [10]. It is based on this premise that the UNICEF designed the EQUitable Impact Sensitive Tool (EQUIST) to help governments and the global health community improve equity in maternal, newborn and child health [11]. EQUIST is an online tool, designed to help health policymakers and program managers to sharpen health plans and policies and make responsible decisions about how to strengthen their health systems. The explicit goal of EQUIST is to reduce health disparities between the most marginalized mothers and young children and the better-off [12].

It is a medium-term strategic planning, modelling and monitoring platform that serves to improve child and maternal health as well as nutrition equity in developing and middle-income countries [11, 12]. Equist, was developed in partnership with the Community Systems Foundation (CSF), with funding from the Bill and Melinda Gates Foundation and builds on previously-existing platforms including the Lives Saved Tool (John Hopkins Bloomberg School of Public Health) and the Marginal Budgeting for Bottleneck tool (a cost estimator used by the World Bank) and on globally available data including demographic health surveys DHS [11]. A key difference between EQUIST and previous tools is that EQUIST is considerably simpler and more user-friendly, with most of the calculations happening automatically [12]. EQUIST contains three modules: situation analysis, scenario development using bottleneck analysis and cost and impact projections [11]. Incorporating EQUIST in the knowledge translation (KT) process will definitely improve the evidence to policy link. The Canada Institutes of Health Research (CIHR) had earlier defined KT as a dynamic and iterative process that includes synthesis, dissemination, exchange and ethically sound application of knowledge to improve the health of people, provide more effective health services and products and strengthen the health care system [13]. A research team with a robust KT competence will be able to use research to inspire stakeholders to think and/or act differently because the KT process is achieved through transmission and exchange of reliable information and through extensive dialogue between the producers and users of the research [14]. EQUIST is therefore is very critical to both KT and evidence-informed policy processes as it can provide very reliable information for example to calculate the impacts and costs of intervention strategies as well as the reduction of disparities in access to and quality coverage of, essential health services [1]. Despite the importance of EQUIST to the evidence informed policymaking process, the knowledge and capacity to use the tool are essentially lacking among policymakers and health researchers in Nigeria. This report is the first EQUIST training workshop in Nigeria.

## Workshop report

**Aim of the workshop**: The aim of this study was to improve the knowledge and capacity of Nigerian MNCH implementation research team (IRT) and policymakers to use EQUIST and KT to promote evidence-informed policymaking in MNCH. This was achieved via a training workshop on EQUIST and KT. A modified “before and after” intervention study design was used in which outcomes were measured on the eligible population (target participants) both before the programme (intervention) is implemented and after [15]. The difference between the before and after measurements was taken to be the impact of the intervention. (In this instance, the “before”- or “baseline”- measurements served as the control measurements).

**Study area and participants:** This study was conducted in Benin City the capital of Edo state in southern Nigeria. The target participants included members of IDRC/WAHO supported IRT of Edo state; policymakers from Edo State Ministry of Health and related agencies; Staff from local government areas health departments; staff of the primary health care development agency (PHCDA); and representatives of the civil society organizations/non-governmental organizations (CSOs/NGOs) as well as media representatives. All the participants were key stakeholders in the IDRC/WAHO supported project entitled: *"Increasing Women's Access to Skilled Care to Reduce Maternal and Perinatal Mortality in Nigeria"* undertaken in Edo state.

**Ethical consideration**: Approval for this study was obtained from the University Research Ethics Committee of Ebonyi State University Nigeria (the institution of the principal author). The approval was based on the agreement that participation in the research was voluntary following informed consent; that participants' anonymity would be maintained; and that every finding would be treated with utmost confidentiality and for the purpose of this research. These were adhered to in this study.

**Facilitators**: The workshop was facilitated by two professors and a senior policymaker. One of the professors has expertise on subject of knowledge translation and the use of EQUIST and is the national consultant for the WAHO MEP Project Nigeria. The other professor has expertise on obstetrics and gynecology and is the principal investigator for the MEP project in Edo state Nigeria and the leader of the IRT. The senior policymaker has vast experience working with the government in the formulation of policy in the south-eastern Nigeria and is also a visiting lecturer at the African Institute for Health Policy & Health Systems, Ebonyi State University Nigeria.

**Pre-workshop tasks**: Up to 45 participants residing within Edo state who are considered stakeholders in maternal and child health issues were mapped out for this study. A three-day intensive health-policy based knowledge translation and use of EQUIST training workshop was held at the Women's Health and Action Research Centre (WHARC), Benin City Nigeria, for the participants. All the participants were invited to the workshop by invitation letters which were sent few weeks before the event and was followed-up with a text message reminder to their mobile phones a day before the programme.

**Programme**: The three-day workshop took place in May 2017. The duration of the workshop each day was nine hours from 8am-4pm (with intermittent tea and lunch breaks). A pre-workshop assessment questionnaire (developed in a 5-point likert scale according to the degree of adequacy; 1 = grossly inadequate, 5 = very adequate), was administered prior to actual training to assess the level of knowledge and capacity of the participants on the specific topics to be covered within the theme of the workshop. After the administration of the pre-workshop questionnaire the training commenced and was facilitated by the resource persons. The workshop covered the following topics: (i) Introduction to health policy & health systems; (ii) Knowledge translation models, measures & tools; (iii) Inter-sectoral collaboration in policymaking & implementation; (iv) Managing political interference in policy making & implementation; (v) Policy review, analysis and contextualization; (vi) Policy formulation and legislation process; (vii) Introduction to MBB: its weakness & replacement by EQUIST; (viii) EQUIST Overview & Theory of change; (ix) EQUIST's situation analysis; (x) EQUIST's scenario analysis; (xi) EQUIST's scenario comparison. The following materials were used for the training workshop session on EQUIST: UNICEF Health Systems Strengthening Approach document [**1**]; EQUIST: Equitable Strategies to Save Lives Technical Notes; EQUIST: Equitable Impact Sensitive Tool-Analyst & General User; EQUIST Instructional Video.

### EQUIST's structure modules: situation analysis, scenario analysis and scenario comparison

**Situation analysis**: The situation analysis module provided to the participants an overview of the health situation in Nigeria, such as health and nutrition outcomes (under-five mortality; neonatal mortality; malnutrition etc), epidemiological components (under-five diarrhoea; pneumonia etc) and coverage of health interventions (antenatal care and skilled birth attendance). Overall, the situation analysis module allowed the participants to visualize the most deprived populations in Nigeria by geographic area and wealth quintile, using maps and bar charts.

**Scenario analysis**: The scenario analysis module was used to teach the participants how to go through the seven-step theory of change by creating a scenario, selecting target populations, epidemiological priorities for these populations and interventions to address these priorities. The impact and cost of the interventions were displayed at the end of the process and participants determined the lives saved. The participants determined the initial three steps, informed by the situation analysis, which included the selection of targeted populations, the identification of the most important health issues for these groups and the prioritization of interventions. Participants then selected the causes of bottlenecks and interventions to address them.

**Scenario comparison**: The participants were taught how to use the scenario comparison to compare the impacts of various user-created scenarios to identify the most cost-effective options for addressing bottlenecks and improving high impact intervention coverage rates. They were taught how they can specifically determine which interventions are more effective in terms of how many lives were saved per unit of financial expenditure. This enabled them decide the most feasible actions to prioritize within certain contexts. All teaching sessions were done using power-point presentation and handouts on each topic were produced and distributed to all participants. It was made mandatory for all lectures to be delivered in simplified, practical and easily comprehensible patterns. Practical sessions were held during the workshop in which each participant was able to use an internet connected computer to practice the use of EQUIST to perform situation analysis, scenario analysis and scenario comparison. At the end of the workshop, a post-workshop assessment questionnaire was administered to the participants to evaluate the impact of the workshop.

**Evaluation**: The data collected via the questionnaire (developed in a likert scale format) was analyzed using the methods developed at McMaster University Canada by Johnson and Lavis [16]. The analysis is based on mean rating (MNR). For instance, the figures represent Likert rating scale of 1-5 points, where 1point = grossly inadequate; 2 points = inadequate; 3 points = fairly adequate; 4 points = adequate and 5 points = very adequate. In terms of analysis, values ranging from 1.00-2.99 points were considered low, whereas values ranging from 3.00-5.00 points considered high. The Pre-workshop means were compared to the Post-workshop means.

**Outcomes**: The pre-workshop mean of understanding and capacity for use of KT ranged from 2.02-3.41, while the post-workshop mean ranged from 3.24- 4.30 on the 5 point scale. The pre-workshop mean of understanding and capacity for use of EQUIST ranged from 1.66-2.41, while the post-workshop mean ranged from 3.56-4.54 on the 5point scale. The percentage increase in mean of KT at the end of the workshop ranged from 8.0%-88.1%, while those of EQUIST ranged from 65.6%-158.4%. Out of the 45 participants invited for the workshop, a total of 38 (84.4%) signed the informed consent form and completed the questionnaire. The profile and official designation attributes as indicated in Table 1 included the following: Females (56.2%), age group 25-34years old (41.2%); Participants from ministry of health and its agencies (44.4%); Researchers (36.1%). A total of 21.1% and 42.1% of the participants had bachelors and masters degrees as highest academic qualifications respectively. The outcome of the assessment of the impact of the training workshop with the comparison of the pre-workshop mean and post-workshop mean is presented in Table 2, Table 3. Result showed a progressive increase in the post-workshop mean over the pre-workshop mean. The summary of the pre-workshop and post-workshop assessment of the knowledge translation concepts is shown in Table 2. The pre-workshop mean of understanding and capacity for use of KT ranged from 2.02-3.41, while the post-workshop mean ranged from 3.24- 4.30 on the 5 point scale. The percentage increase in mean of KT at the end of the workshop ranged from 8.0%-88.1%. In terms of the Introduction to health policy & health systems module, the percentage mean increase ranged from 8.0%-73.7%. Knowledge translation models, measures & tools, percentage mean increase ranged from 48.4%-88.1%, while percentage mean increase of Inter-sectoral collaboration in policymaking & implementation module, ranged from 21.7%-48.4%. Policy review, analysis and contextualization module, recorded percentage mean increase ranging from 37.3%-57.1%. The percentage mean increase of Policy formulation and legislation process module, ranged from 63.1%- 72.2% (Table 2). The summary of the pre-workshop and post-workshop assessment of EQUIST is presented in Table 3. The pre-workshop mean of understanding and capacity for use of EQUIST ranged from 1.66-2.41, while the post-workshop mean ranged from 3.56-4.54 on the 5 point scale. The percentage increase in mean of EQUIST ranged from 65.6%-158.4%. In terms of the Introduction to MBB: its weakness & replacement by EQUIST module, percentage mean increase ranged from 64.2%-97.7%. EQUIST overview & theory of change module, percentage mean increase ranged from 104.0%-130.5%, while percentage mean increase of EQUIST use module, ranged from 126.7%-158.4%.

**Table 1:** Profile and official designation attributes of participants who completed the questionnaire at the workshop

Parameter assessed	Frequency (%)
**Gender**	
Male	14(43.8)
Female	18(56.2)
Total	32
**Age Category**	
25-34years	14(41.2)
35-44years	12(35.3)
>44years	8(23.5)
Total	36
** Type of organization**	
MOH/Govt Agency	16(44.4)
LGA/PHCDA	5(13.9)
NGOs/CSOs	
Researchers	2(5.6)
13(36.1)	
Total	36
**Highest academic qualification**	
Diploma	2(5.3)
Bachelor	8(21.1)
MBBS	4(10.5)
Masters	16(42.1)
Doctorate	6(15.8)
Others	2(5.3)
Total	38

MOH=Ministry of health; Govt =Government; LGA=Local government area, PHCDA=Primary health care development agency; NGOs/CSOs=non-governmental organizations/civil society organizations; MBBS=bachelor of medicine & surgery

**Table 2:** Outcome of the pre-workshop and post workshop questionnaire analysis on knowledge translation concepts at the training workshop in Benin Nigeria

Parameters assessed	Pre-workshop mean	Post-workshop mean	Mean increase	% Mean increase
**Introduction to health policy & health systems**				
Knowledge of the meaning of policy and policy cycle	3.24	3.50	0.26	8.0
Understanding of the critical policy issues and the focus/forms of policy analysis	2.74	3.91	1.17	42.7
Understanding of building blocks of the health systems	3.23	3.97	0.74	22.9
Understanding of Integrated Knowledge Translation (iKT) and End-of-Grant Knowledge Translation (eKT)	2.13	3.70	1.57	73.7
**Knowledge translation models, measures & tools**				
Knowledge of the meaning and core principles of knowledge translation	2.58	3.83	1.25	48.4
Understanding of the four models of knowledge translation	2.02	3.80	1.78	88.1
Knowledge of the Characteristics of Knowledge Translation	2.24	3.68	1.44	64.3
Understanding of the Frameworks Applicable to Knowledge Translation	2.03	3.65	1.62	79.8
Understanding of Knowledge Management and the Strategies	2.31	3.71	1.40	60.6
Understanding of the tools for knowledge translation and exchange	2.21	3.59	1.38	38.4
Knowledge of the preparation and key ingredients of effective policy brief	2.34	3.59	1.25	53.4
Understanding of the need and characteristics of policy dialogue	2.58	3.68	1.10	42.6
**Inter-sectoral collaboration in policymaking & implementation**				
Knowledge of the meaning of inter-sectoral collaboration in policymaking & implementation	2.87	4.26	1.39	48.4
Understanding of what makes collaboration work	3.05	4.12	1.07	35.1
Understanding of the roadblocks to effective collaboration	2.95	4.24	1.29	
**Managing political interference in policy making & implementation**				
Understanding of the political nature of health	3.41	4.15	0.74	21.7
Knowledge about why health has been apolitical	2.84	4.15	1.31	46.1
Understanding of political interference in policymaking and implementation	3.08	4.30	1.22	39.6
Knowledge about managing political interference in policymaking and implementation	2.37	4.06	1.69	71.3
**Policy review, analysis and contextualization**				
Knowledge of the policy review, analysis and contextualization process	2.49	3.91	1.42	57.1
Understanding of the objective of policy review, analysis and contextualization	2.36	3.24	0.88	37.3
Understanding of health systems guidance contextualization framework	2.94	3.73	1.24	42.2
**Policy formulation and legislation process**				
Knowledge of the health policy making models	2.32	3.82	1.50	64.6
Understanding of the stages of the policy cycle and their interrelationship	2.27	3.91	1.64	72.2
Understanding of policy legislation process in Nigeria	2.49	4.06	1.57	63.1

**Table 3:** Outcome of the pre-workshop and post workshop questionnaire analysis on MBB & EQUIST concepts at the training workshop in Benin Nigeria

Parameters assessed	Pre-workshop mean	Post-workshop mean	Mean increase	% Mean increase
**Introduction to MBB: its weakness & replacement by EQUIST**				
Understanding of Marginal Budgeting for Bottlenecks (MBB) as a results-based planning and budgeting tool	2.40	4.21	1.81	75.4
Knowledge of the concept of Marginal Budgeting for Bottlenecks as a tool that identifies implementation constraints of the health system	2.31	4.33	2.02	87.4
Knowledge of the five key steps of the Marginal Budgeting for Bottlenecks	2.41	4.08	1.67	69.3
Knowledge of the three modules of Marginal Budgeting for Bottlenecks	2.26	3.71	1.45	64.2
Knowledge of the description of Marginal Budgeting for Bottlenecks Intervention Packages	2.18	4.12	1.94	89.0
Knowledge about the MBB tool and process for Identifying the Bottlenecks and the determinants	2.10	4.00	1.90	90.5
Knowledge of the weaknesses and limitations of Marginal Budgeting for Bottlenecks	2.15	3.56	1.41	65.6
Knowledge of why MBB has been replaced with Equitable Impact Sensitive Tool (EQUIST)	2.15	4.25	2.10	97.7
**EQUIST Overview & Theory of change**				
Knowledge of the Equitable Impact Sensitive Tool (EQUIST) as a medium-term analysis and strategic planning tool	2.00	4.08	2.08	104.0
Knowledge of Who EQUIST is designed for	1.97	4.38	2.41	122.3
Knowledge of what to expect when using EQUIST	1.97	4.54	2.57	130.5
Understanding of EQUIST Theory of change	1.94	4.17	2.23	114.9
**EQUIST use**				
Knowledge of how EQUIST is used	1.91	4.33	2.42	126.7
Understanding of use of EQUIST to perform situational analysis	1.72	4.33	2.61	151.7
Understanding of use of EQUIST to perform Scenario analysis	1.69	4.33	2.64	156.2
Understanding of use of EQUIST to perform Scenario Comparison	1.66	4.29	2.63	158.4

## Conclusion

The results of this study showed a tremendous improvement in the understanding of the participants regarding the use of knowledge translation and EQUIST to promote MNCH evidence informed policymaking process. An interesting aspect of this study was participant composition, where MNCH policymakers and researchers came together to be trained in order to adequately execute a project that is designed to increase women's access to skilled care to reduce maternal and perinatal mortality. Platforms bringing both policymakers and researchers to consider issues around research-policy interface have been shown to facilitate the uptake of research into policy especially when capacity enhancement mechanisms like training workshops are used [17, 18]. In a recent assessment of policymaker's engagement initiatives to promote evidence informed health policy making in Nigeria, all the 11 studies reviewed which used workshops as strategy recorded positive impacts in relation to quantifiable improvement in policymakers' knowledge and competence in evidence to policy process [19]. In this study, the knowledge translation module conprised six important topics including introduction to health policy & health systems; KT models, measures & tools; inter-sectoral collaboration in policymaking & implementation; managing political interference in policy making & implementation; policy review, analysis and contextualization; and policy formulation and legislation process. These topics were deliberately included in the module to enable the participants to gain a better understanding of the complex nature of the policy making process. Green and Bennett [20] had argued that policy-making does not take place in a vacuum but political, economic and social factors all affect how policies are made, and who makes them, at all levels. According to Jones and Walsh [21], the integration of evidence into policy decision making is a complex process of multiple, frequently competing and/or intertwined sets of influences in which evidence plays just one of many roles.

A major milestone in this study was the introduction of EQUIST as an important tool to help the participants especially the policymakers and program managers to sharpen health plans and policies and make responsible decisions about how to strengthen the health systems [12]. This study was the very first report of EQUIST training workshop in Nigeria. It is worthy of note to state that of all the aspects of this training, the improvement in the participants' understanding of the EQUIST module was tremendous and ranged from 104.0% to 158.4%. Interestingly, the EQUIST enabled the participants to create an accurate picture of the health status of the most deprived children and women in Nigeria, identify which populations are at greatest risk, why they are at risk and how many lives can be saved with appropriate action. This is one of the most important benefits of EQUIST [11]. In addition to this, the tool also helped the participants to practice how to confidently plan health interventions by identifying the highest impact, most cost-effective strategies to level disparities and project the impact of health systems strategies and measure the potential effects in terms of lives saved and costs [11]. The improved understanding of knowledge translation and EQUIST gained by the participants in this study will undoubtedly impact positively on the evidence-to-policy process regarding the MNCH outcomes. A major limitation of this study was the limited duration of the workshop which made adequate impact assessment not to be possible. To assess the real impact of the training, a follow-up may be necessary to assess the extent the participants will use the skill acquired in the policymaking process. This limitation notwithstanding, this workshop is an important first step to bridging the divide between research and policy and understanding how to address equity concerns in the MNCH interventions in Nigeria.

## Competing interests

Authors declare no competing interests.
